# The Associations of Psychological Stress with Depressive and Anxiety Symptoms among Chinese Bladder and Renal Cancer Patients: The Mediating Role of Resilience

**DOI:** 10.1371/journal.pone.0154729

**Published:** 2016-04-29

**Authors:** Mengyao Li, Lie Wang

**Affiliations:** Department of Social Medicine, School of Public Health, China Medical University, No.77 Puhe Road, Shenyang North New Area, Shenyang, 110122, PR China; Vanderbilt University, UNITED STATES

## Abstract

**Background:**

The prevalence of depressive and anxiety symptoms and their associated factors in bladder and renal cancer patients are not well evaluated in China. Given the growing attention to positive psychological constructs in the field of oncology, it is necessary to explore the effects of these constructs on depressive and anxiety symptoms. This study aims to explore the associations of psychological stress with depressive and anxiety symptoms among Chinese bladder and renal cancer patients and the mediating role of resilience in these relationships.

**Methods:**

A cross-sectional study was conducted at the First Affiliated Hospital of China Medical University in Liaoning province. 327 bladder cancer patients and 268 renal cancer patients completed questionnaires on demographic variables, the Center for Epidemiologic Studies Depression Scale, Zung Self-Rating Anxiety Scale, Resilience Scale-14, and Perceived Stress Scale-10 during the period from July 2013 to July 2014. Hierarchical linear regression analyses were performed to explore the mediating role of resilience.

**Results:**

The prevalence of depressive and anxiety symptoms was 78.0% and 71.3% in bladder cancer patients, and 77.6% and 68.3% in renal cancer patients. Psychological stress was positively related to depressive and anxiety symptoms, while resilience was negatively related to these symptoms. Resilience partially mediated the relations of psychological stress with depressive and anxiety symptoms.

**Conclusions:**

The high prevalence of depressive and anxiety symptoms among Chinese bladder and renal cancer patients should receive more attention from medical institutions and government agencies. In addition to reducing depressive and anxiety symptoms, resilience development should be included in depression and anxiety prevention and treatment strategies in China.

## Introduction

Cancer is a complicated disease that often results in death. It has negative effects on physical, psychological, social, and economic well-being of patients. A diagnosis of cancer can exert profound impacts on patients`psychological functioning, not only in the initial period after diagnosis, but also many years thereafter [1, 2]. Some cancer survivors find it difficult to cope with psychosocial problems of cancer, to deal with mental problems such as terror of death, hopelessness, isolation, meaninglessness, and threats to self-identity [3]. Prior research indicated that depression and anxiety were two main forms of psychological distress in cancer patients [4–7]. In oncological and haematological settings, all types of depression occurred in 20.7% (12.9–29.8) of the patients, while the prevalence of anxiety was 10.3% (5.1–17.0) [4]. In China, the morbidities of depression (54.90%) and anxiety (49.69%) were significantly higher among cancer adults than normal adults (depression: 17.50%, anxiety: 18.37%) [8].

Besides the general impacts of cancer diagnosis and treatment, bladder and renal cancer patients have some noticeable differences in terms of social and psychological conditions. Surgery is one of the most common treatments for bladder and renal cancers, and the surgery often involves urinary diversion and urinary tract reconstruction. For instance, after cystectomy and ileostomy, patients need to wear urine bag and use artificial bladder for life, which may bring patients tremendous pain to their physiology, psychology and social interactions.

Psychological stress is defined as the cognitive, behavioral, psychological and physiological reactions experienced when an individual faces a real or virtual situation, in which the demands go beyond one’s psychological endurance [9]. Lazarus et al. has indicated that if an event or a situation is considered stressful, it must work through cognitive processes [10]. The rising levels of stress can have harmful effects on one’s psychology, such as helplessness, sleeplessness, rumination, isolation, irascibility, triggering or aggravating anxiety and depression [9, 11]. This will probably accelerate the development and progression of malignant diseases [12, 13].

Recent research has gradually shifted attention from negative predictors to exploration of the effects of positive constructs in cancer patients [11, 14, 15]. Promoting positive psychosocial outcomes is just as critical as minimizing negative ones. Recent evidence has suggested that positive beliefs and emotions seem not only to be associated with good outcomes among patients experiencing life-threatening illnesses, but also to play a vital role in realizing them [16]. In oncology, several interventions integrating positive psychology factors have shown encouraging preliminary results [17, 18]. In light of the literature review, resilience is a research focus in this field.

By definition, resilience refers to the dynamic capacity of someone to bounce back from life adversities (failures) to maintain or recuperate his psychological health successfully [19]. It is a unique trajectory or mechanism of positive adaptation that changes over time and protects against psychological adversity [20]. Resilience can predict lower levels of distress, better adjustment and health conditions among cancer patients [21, 22]. Hou et al. revealed that patients with high resilience were able to objectively understand their diseases, and more strictly adhere to the treatment to avoid risks and promote protection factors [23]. High resilience also reduces cancer patients`needs for psychosocial support in the face of stressful events (radio-oncological treatment) [24]. Previous research indicated that psychological resilience played a mediating role in the relationships between personal or environmental factors and psychological well-being among cancer patients [21, 25].

Both the associations of psychological stress with depressive and anxiety symptoms and the correlations of resilience with the symptoms have been confirmed by previous studies. Wang et al. identified that psychological stress was a predictor of depressive and anxiety symptoms among cancer patients [26], and Min et al. reported that resilience negatively predicted depressive and anxiety symptoms among metastatic cancer individuals [27]. However, the role of resilience as a mediator in the relationships between psychological stress and depressive or anxiety symptoms among Chinese bladder and renal cancer patients has not been examined to our knowledge. Theoretically, resilience refers to an interactive dynamic construct, a positive coping style and a positive personality trait [20, 27], which can promote more positive psychosocial outcomes in the course of the cancer experience (before, during and after cancer diagnosis) [28]. In light of the above concerns, this paper aims to explore (1) the associations of psychological stress with depressive and anxiety symptoms, and (2) whether resilience mediates the effect of the psychological stress on depressive and anxiety symptoms.

## Materials and Methods

### Ethics statement

This study received ethics approval from the Committee on Human Experimentation of China Medical University. Written informed consent concerning conduct of the survey was obtained from each participant. We protected the privacy of individuals in processing personal data and maintained confidentiality of individual records and accounts.

### Study design and study sample

A cross-sectional study was carried out from July 2013 to July 2014. All participants were from the Department of Urology, the First Affiliated Hospital of China Medical University. Inclusion criteria in this study were as follows: (1) 18 years old or above, (2) had primary school diploma or above, (3) with pathological diagnosis of bladder or renal cancer, (4) spoke Chinese language, (5) were cooperative with treatments. Exclusion criteria included that patients had (1) intellectual impairments, (2) a history of psychiatric problems before cancer diagnosis, (3) other active tumors. Self-administered questionnaires were distributed to the patients after obtaining their informed consents. There were strict quality control measures to avoid possible bias. Each patient was distributed the questionnaire to complete in a private place within one week after surgery. Of the 370 bladder cancer patients and 297 renal cancer patients who met the inclusion criteria, 72 patients were excluded because they declined to participate or the missing values exceeded 30% in the questionnaire. The final effective response rate was 88.38% of bladder cancer patients and 90.24% of renal cancer patients.

### Measurement depressive symptoms

Depressive symptoms were measured with the Center for Epidemiologic Studies Depression Scale (CES-D) [29]. The CES-D scale is comprised of 20 items, and each item includes four responses, ranging from 0 “rarely or none of the time (less than 1 day)” to 3 “most or all of the time (5 to 7 days)”. The summed score ranges from 0 to 60, with a higher score indicating more serious depressive symptoms. The presence of depressive symptoms was defined as a CES-D score≥16. The Chinese version has been widely used in previous studies [30, 31]. The Cronbach’s alpha for the CES-D scale was 0.834 in the present study.

### Measurement anxiety symptoms

Anxiety symptoms were assessed with the Zung Self-Rating Anxiety Scale (SAS) [32]. The SAS scale consists of 20 items, and each item is answered on a 4-point Likert-type scale ranging from “never” to “always”. The raw score is standardized according to the formula: standardized score = int (1.25*raw score). Higher score means more serious anxiety symptoms. The presence of anxiety symptoms was defined as a SAS standardized score≥50. The Chinese version has been widely used in previous studies [31]. In this study, the Cronbach’s alpha for the scale was 0.850.

### Measurement of resilience

The Resilience Scale-14 (RS) was widely used in resilience research [33]. The item is answered using a 7-point Likert-type scale ranging from “strongly disagree” to “strongly agree” [34]. The total score of the scale is calculated to obtain a composite resilience value with higher scores indicating higher levels of resilience. The Chinese version had been used in previous studies, and the reliability and validity had been confirmed [33, 34]. In this present study, the Cronbach’s alpha coefficient for the total scale was 0.950.

### Measurement of psychological stress

Psychological stress was measured with the Perceived Stress Scale-10(PSS-10) [35]. Each question is answered on a 5-point Likert scale ranging from 0 “never” to 4 “very often”. The summed score ranges from 0 to 40, with a higher score indicating higher level of psychological stress. The Chinese version of the scale had demonstrated adequate reliability and validity [36, 37]. In this present study, the Cronbach’s alpha coefficient for the total scale was 0.632.

### Demographic characteristics

Age, education levels, marital status, physical activity, cancer stage, perfusion chemotherapy and DC tumor vaccine were obtained in this study. Age was divided into “18–55 years”, “56–65 years”, and “over 65 years”. Education levels were categorized into “primary/middle school”, “high school” and “junior college and above”. Marital status was categorized into two groups of “single/ divorced/separated/widowed” and “married/cohabitation”. Cancer stages were categorized into “I”, “II” and “III”. Others were divided into two groups of “yes” or “no” groups. In addition, perfusion chemotherapies were only used among bladder cancer patients, and DC tumor vaccines were used among renal cancer patients.

### Statistical analysis

All analyses were performed with SPSS 17.0 program. All statistical tests were two-sided (α = 0.05). Descriptive statistics of the demographic and study variables were indicated with mean, standard deviation (SD), number (N) and percentage (%) as appropriate. T-tests were used to examine the differences of continuous variables in gender, marital status, physical activity, perfusion chemotherapy and DC tumor vaccine. Study variables were compared among age groups, different education levels’ groups and cancer stage groups by one-way ANOVA analyses. When a significant difference was found, least-significant-difference tests (LSDs) were performed for multiple comparisons.

It is a partial mediation if the independent variable (psychological stress) has significant effect on the dependent variable (depressive or anxiety symptoms) when the mediator (resilience) is added [38]. It is a full mediation if the independent variable (psychological stress) has no effect on the dependent variable (depressive or anxiety symptoms) when the mediator (resilience) is added [38].

All continuous variables were centralized to avoid multi-collinearity before the regression analyses [39]. Pearson correlation, linear regression and hierarchical linear regression analyses for depressive and anxiety symptoms were used to examine the mediating effects. In step 1, education levels, cancer stages and physical activity were used as control variables because these study variables differed significantly on depressive and anxiety symptoms. In step 2, psychological stress was entered. In step 3, resilience was added. In addition, the Sobel tests were also used to test the mediation effects.

## Results

### Demographic and clinical characteristics of the studied population and prevalence of depressive and anxiety symptoms

Among bladder cancer patients, demographic and clinical characteristics of the subjects and the distributions of depressive and anxiety symptoms in categorical variables were shown in Table 1. The mean age of bladder cancer patients was 63.77(SD = 11.75) and 80.7% were males. Most participants (63.3%) were diagnosed with stage I cancers. The mean score of depressive symptoms was 23.57 and the prevalence of depressive symptoms was 78.0%. The mean score of anxiety symptoms was 55.27 and the prevalence of anxiety symptoms was 71.3%. About 57.74% of participants had received perfusion chemotherapies. Patients with university degrees had lower levels of depressive and anxiety symptoms than others.

**Table 1:** Demographic and clinical characteristics of subjects and the distributions of depressive and anxiety symptoms among bladder cancer patients.

Variables	N(%)	Depressive symptoms	Anxiety symptoms
		Mean(SD)	Mean(SD)
Gender		p>0.05	p>0.05
Male	264(80.7)	23.62(9.09)	55.56(11.57)
Female	63(19.3)	23.38(10.06)	54.05(12.63)
Age		p>0.05	p>0.05
≤55	71(21.7)	21.75(9.84)	53.96(11.60)
56–65	101(30.9)	23.70(8.50)	55.38(11.78)
≥66	155(47.4)	24.32(9.43)	55.80(11.89)
Marital status		p>0.05	p>0.05
Single/Divorced/Separated/Widowed	41(12.5)	24.27(8.35)	54.67(11.80)
Married/Cohabitation	286(87.5)	23.47(9.40)	55.36(11.80)
Education level		p<0.05	p<0.01
Primary/Middle school	185(56.6)	24.60(8.95)	55.85(11.27)
High school	73(22.3)	24.12(8.32)	56.90(11.17)
Junior College and Over	69(21.1)	20.22(10.36)	52.00(13.22)
Physical Activity		p<0.05	p<0.05
No	184(56.3)	24.74(8.57)	56.70(11.31)
Yes	143(43.7)	22.06(9.93)	53.44(12.15)
Cancer Stage		p>0.05	p>0.05
I	207(63.3)	22.94(9.11)	54.89(11.98)
II	117(35.8)	24.63(9.56)	55.93(11.49)
III	3(0.9)	25.67(7.57)	56.25(12.05)
Perfusion Chemotherapy		p>0.05	p>0.05
No	139(42.51)	23.82(9.18)	56.25(11.61)
Yes	188(57.49)	23.39(9.36)	54.55(11.88)

Among renal cancer patients, demographic and clinical characteristics of the subjects and the distributions of depressive and anxiety symptoms in categorical variables were shown in Table 2. The mean age of renal cancer patients was 57.17(SD = 11.36) and 62.7% were males. Patients diagnosed with stage III cancers had the highest level of anxiety symptoms. The mean score of depressive symptoms was 23.39 and the prevalence of depressive symptoms was 77.6%. The mean score of anxiety symptoms was 55.42 and the prevalence of anxiety symptoms was 68.3%. About 36.94% of them had received DC tumor vaccines.

**Table 2:** Demographic and clinical characteristics of subjects and the distributions of depressive and anxiety symptoms among renal cancer patients.

Variables	N(%)	Depressive symptoms	Anxiety symptoms
		Mean(SD)	Mean(SD)
Gender		p>0.05	p>0.05
Male	168(62.7)	22.71(9.64)	54.59(11.82)
Female	100(37.3)	24.54(8.63)	56.83(10.94)
Age		p>0.05	p>0.05
≤55	114(42.5)	23.32(9.50)	55.02(11.40)
56–65	93(34.7)	23.56(8.39)	56.08(10.92)
≥66	61(22.8)	23.26(10.32)	55.18(12.75)
Marital status		p>0.05	p>0.05
Single/ Divorced/Separated/Widowed	24(9.0)	23.08(10.98)	54.95(12.89)
Married/Cohabitation	244(91.0)	23.42(9.14)	55.47(11.41)
Education level		p<0.01	p<0.01
Primary/Middle school	113(42.2)	24.45(8.83)	57.23(10.70)
High school	78(29.1)	24.85(8.24)	57.64(10.25)
Junior College and Over	77(28.7)	20.36(10.34)	50.52(12.54)
Physical Activity		p<0.01	p<0.01
No	145(54.1)	24.77(8.48)	57.47(10.69)
Yes	123(45.9)	21.76(9.97)	53.02(12.05)
Cancer Stage		p>0.05	p<0.01
I	139(51.9)	22.09(9.94)	53.18(12.06)
II	125(46.6)	24.77(8.40)	57.80(10.52)
III	4(1.5)	25.75(7.89)	59.06(8.50)
DC Tumor Vaccine		p>0.05	p>0.05
No	169(63.06)	23.07(9.79)	55.10(11.80)
Yes	99(36.94)	23.94(8.42)	55.98(11.08)

### Correlations among psychological stress, resilience, depressive and anxiety symptoms

Pearson correlation analyses indicated that psychological stress was positively related to depressive and anxiety symptoms, whereas resilience was negatively related to depressive (-0.509, p<0.01) and anxiety symptoms (-0.499, p<0.01) among bladder cancer patients. And psychological stress was negatively related to resilience (-0.437, p<0.01).

Among renal cancer patients, psychological stress was positively related to depressive and anxiety symptoms, whereas resilience was negatively related with depressive (-0.443, p<0.01) and anxiety symptoms (-0.426, p<0.01). And psychological stress was negatively related to resilience (-0.323, p<0.01).

### Regression analyses with depressive symptoms as the criterion variable

As shown in Table 3, psychological stress was significantly related to depressive symptoms. Psychological stress explained 43.1% of the variance in the model among bladder cancer patients and explained 36.7% of the variance among renal cancer patients. Resilience was negatively and significantly related to depressive symptoms. Resilience accounted for an additional 4.7% of the variance among bladder cancer patients, and accounted for an additional 5.0% of the variance among renal cancer patients. The regression coefficients for psychological stress diminished when resilience was added. Sobel tests also confirmed that resilience had mediating effects in relations between psychological stress and depressive symptoms among bladder (z = 4.84, p<0.01) and renal cancer (z = 6.43, p<0.01) patients.

**Table 3:** Hierarchical linear regression analyses results of depressive symptoms.

Variables	bladder cancer patients			renal cancer patients		
	Step1(β)	Step2(β)	Step3(β)	Step1(β)	Step2(β)	Step3(β)
**Block 1**						
Education1	0.217**	0.124**	0.081	0.204**	0.086	0.045
Education2	0.177**	0.065	0.036	0.199**	0.065	0.022
Stage1	0.075	0.061	0.063	0.119*	0.017	0.005
Stage2	0.015	0.001*	0.007	0.023	-0.001	0.003
Physical activity	-0.129*	-0.045	-0.033	-0.144*	-0.106*	-0.079
**Block 2**						
Psychological stress		0.669**	0.568**		0.630**	0.564**
**Block 3**						
Resilience			-0.245**			-0.243**
F	3.993**	51.211**	52.915**	4.574**	35.157**	36.736**
R^2^	0.059	0.49	0.537	0.08	0.447	0.497
Adjusted R^2^	0.044	0.48	0.527	0.063	0.434	0.484
ΔR^2^	0.059	0.431	0.047	0.08	0.367	0.050

* p<0.05;

** p<0.01

“Education 1” means “primary/middle school” vs. “junior college and over”, “Education 2” means “high /secondary school” vs. “junior college and over”; “Stage 1” means “cancer stage II” vs. “cancer stage I”, “Stage 2” means “cancer stage III” vs. “cancer stage I”

### Regression analyses with anxiety symptoms as the criterion variable

The results in Table 4 also demonstrated that psychological stress was significantly related to anxiety symptoms. Psychological stress explained 41.6% of the variance in the model among bladder cancer patients and explained 27.1% among renal cancer patients. Resilience was negatively and significantly associated with anxiety symptoms. Resilience accounted for an additional 4.8% of the variance among bladder cancer patients and 4.1% among renal cancer patients. The regression coefficients for psychological stress decreased when resilience was added. Sobel tests also confirmed that resilience had mediating effects in relations between psychological stress and anxiety symptoms among bladder (z = 3.91, p<0.01) and renal cancer (z = 3.78, p<0.01) patients.

**Table 4:** Hierarchical linear regression analyses results of anxiety symptoms.

Variables	bladder cancer patients			renal cancer patients		
	Step1(β)	Step2(β)	Step3(β)	Step1(β)	Step2(β)	Step3(β)
**Block 1**						
Education1	0.205**	0.104**	0.063	0.270**	0.167**	0.131*
Education2	0.218**	0.100*	0.065	0.254**	0.136*	0.098
Stage1	0.096*	0.042	0.039	0.169*	0.08	0.069
Stage2	0.017	0	0.005	0.03	0.009	0.013
Physical activity	-0.148**	-0.090**	-0.070*	-0.169*	-0.136**	-0.111*
**Block 2**						
Psychological stress		0.611**	0.526**		0.551**	0.491**
**Block 3**						
Resilience			-0.242**			-0.219**
F	2.740*	44.802**	46.609**	8.112**	30.777**	31.066**
R^2^	0.041	0.457	0.506	0.134	0.414	0.455
Adjusted R^2^	0.026	0.446	0.495	0.118	0.401	0.441
ΔR^2^	0.041	0.416	0.049	0.134	0.280	0.041

* p<0.05;

** p<0.01

“Education 1” means “primary/middle school” vs. “junior college and over”, “Education 2” means “high /secondary school” vs. “junior college and over”; “Stage 1” means “cancer stage II” vs. “cancer stage I”, “Stage 2” means “cancer stage III” vs. “cancer stage I”

## Discussion

This study investigated the prevalence of depressive and anxiety symptoms among Chinese patients with bladder and renal cancers, explored the associations of psychological stress with depressive and anxiety symptoms, and the mediating role of resilience in the relationships. A large sample and a high effective response rate were able to show a good representation and thus enhance the generalizability of our conclusion.

Chinese patients with bladder and renal cancers suffered seriously from depressive and anxiety symptoms. In this research, we found that 78.0% of the Chinese bladder cancer patients had depressive symptoms while 71.3% had anxiety symptoms. And 77.6% of Chinese renal cancer patients had depressive symptoms while 68.3% had anxiety symptoms. The results were the same with the meta-analysis assessing the prevalence of depression (54.90%, range: 20%–89%) and anxiety (49.69%, range: 20%–89.13%) in Chinese cancer adults [8]. This prevalence was much higher than renal or bladder cancer patients in developed countries [40]. For example, Cohen et al. indicated that 23% of patients with metastatic renal cell carcinoma had depressive symptoms in the USA [41]; Henningsohn et al. showed that 35% of bladder cancer patients had depressive symptoms after surgery and 20% of them had anxiety symptoms in Sweden [42]; Ficarra et al. pointed out that about 22% of patients with urological malignant neoplasms(renal, bladder, prostate and penile tumors) had depression symptoms and 14% of them had anxiety symptoms in Italy [43]. These results should be noticed because the prevalence of depression and anxiety in our study was at a high level. There were three possible explanations for the results. First, insufficient knowledge about bladder and renal cancers caused great psychological burden for the patients. Many patients felt overwhelmed by the diagnoses and treatments of cancer, and the medical institutions could not offer professional counseling for cancer patients. Second, education level might be an important influencing factor of depressive and anxiety symptoms. Our results indicated that patients with the highest education level had the lowest levels of depressive and anxiety symptoms. However, 50.1% of participants in our study received less than 10 years of education. Third, urological cancers (bladder and renal cancers) had huge physical (urinary incontinence, sexual dysfunction and poor body image) and related psychological effects, especially for males. The physical functioning impairments can significantly affect their psychological health, such as self-confidence and self-esteem, initiating or aggravating depressive and anxiety symptoms among Chinese bladder and renal cancer patients.

In the present study, we examined the relations of psychological stress with depressive and anxiety symptoms, respectively. Psychological stress was positively correlated with depressive and anxiety symptoms. Patients diagnosed with cancer face severe stressors that may result in psychological distress [44, 45], such as depression and anxiety. They usually experience a series of negative emotions, and some of them have to confront progressive illness and death [9]. The stress triggered by cancer diagnosis directly affects patients’ psychological health. The uncertainty of treatment outcomes may aggravate the patients`mental health, even impact their behaviors [9]. Having the skills to handle complicated clinical conditions could protect patients against becoming excessively concerned about their physical changes, particularly treatment-related changes in urinary or sexual functioning [46].

Recent research indicated that resilience was negatively related to depressive and anxiety symptoms [25, 27]. Resilience may reduce depression and anxiety through fostering some unique characteristics (self-examination, responsibility, tolerance, the ability to adapt to different environmental requirements, and a sound perspective on reality) [47, 48]. Another research showed that resilience could help individuals resist the conversion of past or current anxiety into current depression [25]. These findings contributes to the understanding that resilience has a main effect on depressive and anxiety symptoms and is a positive resource for combating depressive and anxiety symptoms among bladder and renal cancer patients.

This is the first study to examine the role that resilience may play in relations of psychological stress with depressive and anxiety symptoms among Chinese bladder and renal cancer patients. Our findings revealed that resilience partially mediated the effects of psychological stress on depressive and anxiety symptoms. Results in the present study were in accordance with previous studies. Sharpley et al. indicated that psychological resilience was a significant buffer against depression among prostate cancer patients [49]. Patients who perceived higher levels of psychological stress from cancer would have lower levels of resilience which in turn increased the possibilities of developing depressive and anxiety symptoms. Meanwhile, higher level of resilience is conducive to adjustment of cancer patients to their diseases and treatments with positive attitudes [34]. In other words, patients with high resilience are able to maintain stable psychological states, physical and social functioning [50, 51]. Resilience also facilitates better coping with cancer and results in less emotional distress [27]. Compared to relieving patients`psychological distress (psychological stress, depression and anxiety), it is a more positive and feasible strategy for hospitals to develop and promote resilience of cancer patients, in order to improve their emotional health and quality of life in the long run.

## Implications

Findings from our study have practical implications for prevention and treatment of depressive and anxiety symptoms among Chinese bladder and renal cancer patients, because resilience can be fostered and developed through various types of interventions [49]. For example, Caitlin et al. demonstrated that a group-based resilience training intervention was feasible and effective in patients with previous breast cancer [52]. Resilience may manifest at each time point with different clinical characteristics [45]. Interventions aimed at increasing resilience might also be useful for patients with bladder and renal cancers. Promoting resilience is a critical element of patient psychosocial care. Previous research demonstrated that resilience could be effectively developed among prostate cancer patients to decrease depression after their first-diagnoses [49]. Fostering resilience through life span can help cancer survivors to adjust to the challenges of their situations and to resume their daily life [50]. In addition, the high prevalence of depressive and anxiety symptoms in bladder and renal cancer patients should receive high attention from Chinese medical institutions and government agencies. Educating patients about relevant knowledge is another way to relieve their psychological distress. Better knowledge and understanding of their diseases may help patients understand where they stand and cope with cancer more positively. ZHAO et al. demonstrated that continuous nursing intervention combined with health education for kidney cancer patients after radical resection could effectively relieve their negative emotions and enhance quality of life [53]. Further studies could be aimed at exploring whether the psychosocial interventions and cancer-related education based on our study are feasible and effective in bladder and renal cancer patients.

## Limitations

There were several limitations for the present study. Firstly, because it was characterized by cross-sectional design based on self-reported measures, conclusions on the causality of the associations observed among psychological stress, resilience and depressive or anxiety symptoms could not be drawn. Additionally, the interpretation of our results should be made with caution because we did not have a control group, otherwise, the prevalence of depressive and anxiety symptoms in this study could be more reliably and accurately determined. Furthermore, all the subjects were from a single hospital in China, which might limit the generalizability of this study to other regions. Moreover, patients’ knowledge about bladder and renal cancers were not measured. Further studies are needed to validate the current findings. Lastly, further studies need to be conducted to examine whether the results of our study are appropriate in different cultures. These above insufficient points need to be substantiated in future research.

## Conclusions

Chinese bladder and renal cancer patients suffer from severe depressive and anxiety symptoms. Psychological stress was positively related to depressive and anxiety symptoms. Resilience could be a positive psychological resource for combating depressive and anxiety symptoms. More importantly, resilience partially mediated the associations of psychological stress with depressive and anxiety symptoms. It might be important for hospitals to adopt strategies to decrease depressive and anxiety symptoms of bladder and renal cancer patients. Developing programs to enhance patients`resilience seems to be feasible to enhance physical and psychological health of bladder and renal cancer patients in China.

## Supporting Information

S1 Data The S1 Data includes the data underlying our findings in this study. (XLSX)
